# Finger posture modulates structural body representations

**DOI:** 10.1038/srep43019

**Published:** 2017-02-22

**Authors:** Luigi Tamè, Elanah Dransfield, Thomas Quettier, Matthew R. Longo

**Affiliations:** 1 Department of Psychological Sciences, Birkbeck, University of London, London, United Kingdom

## Abstract

Patients with lesions of the left posterior parietal cortex commonly fail in identifying their fingers, a condition known as *finger agnosia*, yet are relatively unimpaired in sensation and skilled action. Such dissociations have traditionally been interpreted as evidence that structural body representations (BSR), such as the *body structural description*, are distinct from sensorimotor representations, such as the *body schema*. We investigated whether performance on tasks commonly used to assess finger agnosia is modulated by changes in hand posture. We used the ‘in between’ test in which participants estimate the number of unstimulated fingers between two touched fingers or a localization task in which participants judge which two fingers were stimulated. Across blocks, the fingers were placed in three levels of splay. Judged finger numerosity was analysed, in Exp. 1 by direct report and in Exp. 2 as the actual number of fingers between the fingers named. In both experiments, judgments were greater when non-adjacent stimulated fingers were positioned far apart compared to when they were close together or touching, whereas judgements were unaltered when adjacent fingers were stimulated. This demonstrates that BSRs are not fixed, but are modulated by the real-time physical distances between body parts.

Knowledge of the spatial configuration of bodies is mediated by a representation called the *body structural description*, damage to which results in conditions such as *autotopoagnosia*^1^,^2^ and *finger agnosia*^3^. Following left parietal lesions, such patients fail to point to body parts on verbal command (autotopoagnosia) or to identify their fingers (finger agnosia), yet may be relatively unimpaired in skilled action^4^. For example, a patient described by Sirigu and colleagues^2^ was unable to answer questions assessing knowledge of the spatial relations between body parts, such as “is the wrist next to the forearm?”, but could answer questions assessing functional knowledge about body parts, such as “what are the eyes for?”. In finger agnosia, patients are impaired at tasks that require identification of the fingers, especially by naming. A typical task for assessing finger gnosis is the “in-between task”, in which participants estimate the number of unstimulated fingers in-between two touched fingers^3^. In order to solve this task, the participant has to perform at least two processing stages: (1) identifying which fingers are touched, and (2) locating the touched fingers within a structural model of the hand that represents at least the touched fingers and the untouched fingers^5^. Therefore, this complex coding processing cannot be solved solely using sensory representations, but requires the use of higher-level body structural representations. Studies of neurological patients^6^ and healthy adults^7^,^8^,^9^ have converged in showing that the left and right parietal cortices may mediate the structural representations of the body (BSR), though the contribution of the two hemispheres may differ qualitatively. A study by Rusconi and colleagues, using a bi-manual version of the in-between task, suggests that the connections between the left anteromedial inferior parietal lobe (a-mIPL) and the precuneus (PCN) provide the core substrate of an explicit bilateral BSR for the fingers that when disrupted can produce the typical symptoms of finger agnosia^9^, compared to the bilateral posterior parietal cortex that contributes to on-line sensorimotor representations^10^.

Such dissociations have traditionally been interpreted as evidence that structural representations of the body are distinct from sensorimotor representations, such as the body schema^6^,^11^,^12^,^13^. The body schema is a dynamic representation of body position which operates outside of conscious awareness to guide and control skilled action^14^,^15^. For example, Castiello and colleagues^16^ have shown that when participants were asked to reach for visual objects which were suddenly displaced after reach onset, they corrected their reach trajectory more than 300 ms before they were consciously aware of the displacement^16^. By contrast, the body structural description seems not to be affected by on-line sensorimotor representations of the body. For instance, an autotopagnosic patient (G.L.) who performed poorly when asked to point or identify his own or other people’s body parts, nevertheless showed normal preparatory grips necessary to grasp objects^4^. In healthy humans, Rusconi and colleagues^5^ provided behavioural evidence in favour of the existence of body structural representations involving an allocentric representation of finger order, independent of hand posture^5^. This division makes intuitive sense, since while body posture may change rapidly moment-to-moment, overall body structure is highly stable. The neural correlates of such dissociations have been recently investigated in an analytic meta-analysis suggesting a selective involvement of the primary somatosensory cortex and the supramarginal gyrus in mediating non-action-oriented body representations. In contrast, other areas such as the primary motor cortex and the right extrastriate body area appear to mediate body representations that support actions^17^. However, knowledge of the spatial relations between body parts, and particularly between the fingers, may play a role in the production of motor responses (e.g., finely tuned movements), therefore, these representations could potentially be less fixed than is commonly believed.

With this idea in mind, we investigated whether structural body representations are modulated by changes in body posture. We tested healthy participants in two classic tests used to assess finger agnosia, the “in-between” test and a tactile localization task, to determine whether structural body representations vary as a function of finger posture. Across blocks, the hands were placed in three postures: (1) fingers touching each other, (2) fingertips separated by one centimetre, and (3) fingers spread to the maximum comfortable splay. Participants judged the numbers of unstimulated fingers “in between” the two touched fingers (Experiment 1) or verbally identified which two fingers had been touched (Experiment 2). We measured whether perceived finger numerosity is altered by changes in the external spatial relations among the fingers. Several potential sources of top-down and bottom-up information^18^, as well as motor-functional features^19^ (i.e., body parts with relative different significance for action and cognition such as hand and foot), have been proposed to contribute to structural body representations. For instance, some authors have suggested that it derives primarily from visual inputs that define body part boundaries and proximity relationships^6^. Note that, unlike these previous works, in the present study we focused on the contribution of touch and postural information in generating structural body representations, while visual information was not manipulated.

## Results

### Experiment 1: in-between test

**Figure 1:**
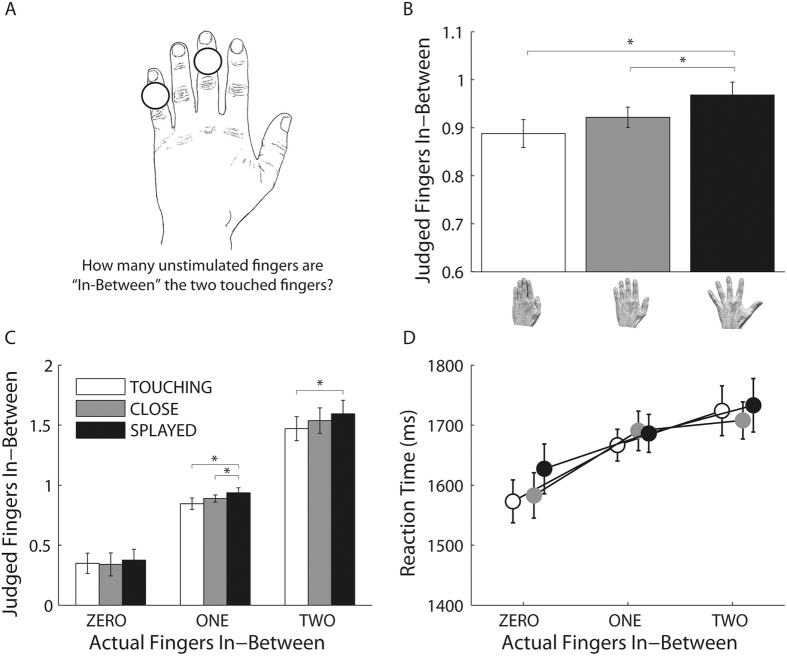
Schematic representation of the in-between task (**A**) performed by the participants for the condition in which one finger was present in-between the two simultaneously stimulated fingers. In the example, the fingers were separated by one centimetre. Judged fingers numerosity as a function of posture (i.e., fingers touching, fingers close at 1 cm and fingers splayed) (**B**) and judged fingers numerosity for the different number of fingers in-between as a function of posture (**C**). Reaction Times (RTs) for the different number of fingers in-between as a function of posture (i.e., fingers touching, fingers close at 1 cm and fingers splayed) (**D**). Error bars indicate 95% Confidence Intervals of the within participants variability (95%CI). *Denotes P < 0.05.

**Figure 2:**
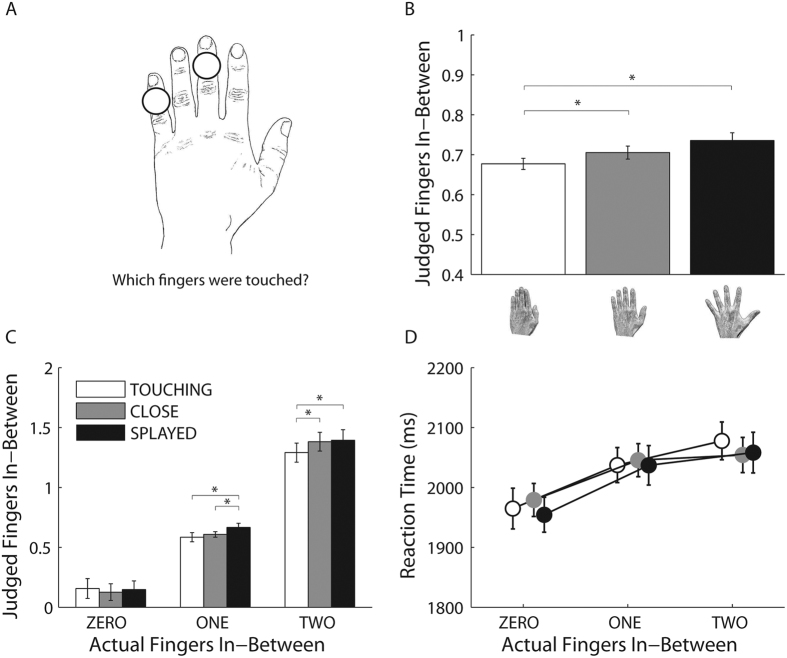
Schematic representation of the localization task (**A**) performed by the participants for the condition in which one finger was present in-between the two simultaneously stimulated fingers. In the example, the fingers were separated by one centimetre. Judged fingers numerosity as a function of posture (i.e., fingers touching, fingers close at 1 cm and fingers splayed) (**B**) and judged fingers numerosity for the different number of fingers in-between as a function of posture (**C**). Note that the judged number of fingers in-between was obtained indirectly by calculating the number of fingers in between the two fingers that the participant judged to have been stimulated. Reaction Times (RTs) for the different number of fingers in-between as a function of posture (i.e., fingers touching, fingers close at 1 cm and fingers splayed) (**D**). Error bars indicate 95% Confidence Intervals of the within participants variability (95%CI). *Denotes P < 0.05.

Figure 1C shows judged finger numerosity for the three in-between fingers conditions as a function of hand posture for Experiment 1. There was a main effect of fingers-inbetween, *F*(2,58) = 149.94, *p* < 0.0001, MSE = 0.201, η_p_^2^ = 0.84, showing, unsurprisingly, that judged numerosity increased monotonically with actual numerosity. Critically, posture also modulated the perceived numerosity of fingers in-between the stimulated fingers, *F*(2,58) = 6.23, *p* < 0.004, MSE = 0.024, η_p_^2^ = 0.18 (Fig. 1B). Numerosity judgments were higher when the fingers were splayed (M ± SE = 0.97 ± 0.05) than when they were close (M ± SE = 0.92 ± 0.05, *t*(29) = 2.36, *p* < 0.025, *d*_*z*_ = 0.43) or touching (M ± SE = 0.87 ± 0.06, *t*(29) = 3.05, *p* < 0.005, *d*_*z*_ = 0.56). The close and touching conditions did not differ from each other, *t*(29) = 1.53, *p* > 0.14, *d*_*z*_ = 0.28. This postural effect was modulated by the number of fingers in-between, *F*(4,116) = 2.77, *p* < 0.031, MSE = 0.007, η_p_^2^ = 0.09. For clarity, we ran three separate one-way ANOVAs with position as within-participants factor, one for each number of actual fingers in-between (i.e., Zero, One, Two). When zero fingers were in-between, there was no significant main effect of posture (*F*(2,58) = 1.26, *p* > 0.292, MSE = 0.008, η_p_^2^ = 0.04). In contrast, when there was one finger in-between, there was a main effect of posture, *F*(2,58) = 6.01, *p* < 0.004, MSE = 0.010, η_p_^2^ = 0.17, with higher numerosity judgments when the fingers were splayed (M ± SE = 0.94 ± .06) than when they were close (M ± SE = 0.89 ± .05, *t*(29) = 2.35, p < 0.03, *d*_*z*_ = 0.43) or touching (M ± SE = 0.85 ± .06, *t*(29) = 2.84, p < 0.008, *d*_*z*_ = 0.52). A main effect of posture was also present when there were two fingers in-between, *F*(2,58) = 5.90, *p* < 0.005, MSE = 0.019, η_p_^2^ = 0.17, caused by higher numerosity judgments when the fingers were splayed (M ± SE = 1.59 ± .06) than when they were touching (M ± SE = 1.47 ± .06, *t*(29) = 3.28, p < 0.003, *d*_*z*_ = 0.60). Note that when there were no fingers in-between perfect performance (i.e., 100 percent correct) would result in a mean value of 0, whereas when there was one finger in-between perfect performance would give a mean of 1, and when there were two fingers in-between a mean of 2 (Figs 1 and 2).

As shown in Fig. 1D analysis of RT revealed no main effect of posture, *F*(2,58) = 1.46, p > 0.23, MSE = 12579.260, η_p_^2^ = 0.05, nor interactions, *F*(4,116) = 2.13, p > 0.08, MSE = 3548.880, η_p_^2^ = 0.07. This shows that the effects we observe cannot be explained by the task simply being easier with the fingers splayed, nor in terms of a speed-accuracy trade-off. There was a significant main effect of finger in-between, *F*(2,58) = 14.96, p < 0.0001, MSE = 24219.394, η_p_^2^ = 0.34, with RTs increasing monotonically with the number of fingers in-between. Results of this experiment support the notion that the body structural representations are not fix as commonly thought, but instead vary as a function of the relative position of the body.

### Experiment 2: localization task

There are two obvious interpretations of the results of Experiment 1. Changes in posture might have altered the representation of the fingers themselves. For example, pressing the fingers together might lead to the disappearance or merging of finger representations. Alternately, posture may have altered the localisation of touch, leading to differences in which fingers were perceived as stimulated in the different postures. Either of these possibilities might have led to differences in judgments of the number of fingers in-between the stimulated fingers.

To address this question we used a tactile localization task in which a different group of participants verbally judged which two fingers were touched. Like the in-between test, the tactile localization task is thought to reflect higher level processing of finger gnosis^13^, however, in this task participants performance will reflect judgments only of the localization of touch and therefore of the representations of the two fingers touched rather than a more extensive part of the hand, including also the fingers in-between the fingers touched. If the results of Experiment 1 derive from the different performance in the localization of the fingers touched, in Experiment 2 we should expect a similar profile to Experiment 1, with modulatory effect in the fingers numerosity estimate as a function of the physical distance between the fingers.

Figure 2C shows judged finger numerosity as a function of hand posture for Experiment 2. There was a main effect of fingers-inbetween, *F*(2,58) = 258.31, *p* < 0.0001, MSE = 0.130, η_p_^2^ = 0.90, showing again that judged numerosity increased monotonically with actual numerosity. Critically, posture modulated the perceived numerosity of fingers in-between also when participants have to localise the stimulated fingers, *F*(2,58) = 7.87, *p* < 0.001, MSE = 0.01, η_p_^2^ = 0.21 (Fig. 2B). Numerosity judgments were lower when the fingers were touching (M ± SE = 0.68 ± 0.02) than when they were close (M ± SE = 0.71 ± 0.03, *t*(29) = 2.35, *p* < 0.026, *d*_*z*_ = 0.43) or splayed (M ± SE = 0.74 ± 0.03, *t*(29) = 3.87, *p* < 0.001, *d*_*z*_ = 0.71). The close and splayed conditions did not differ from each other, *t*(29) = 1.81, *p* > 0.08, *d*_*z*_ = 0.33.

As for the in-between task in Experiment 1, this postural effect was modulated by the number of fingers in-between, *F*(4,116) = 4.31, *p* < 0.003, MSE = 0.009, η_p_^2^ = 0.13. As above, we ran three separated one-way ANOVAs with position as within-participants factor, one for each number of fingers in-between. When there were zero fingers in-between there was no significant main effect of posture (*F*(2,58) = 0.98, *p* > 0.383, MSE = 0.007, η_p_^2^ = 0.03). In contrast, when there was one finger in-between there was a main effect of posture, *F*(2,58) = 7.28, *p* < 0.002, MSE = 0.007, η_p_^2^ = 0.20, with higher numerosity judgments when the fingers were splayed (M ± SE = 0.67 ± 0.03) than when they were close (M ± SE = 0.61 ± 0.03, *t*(29) = 2.64, p < 0.01, *d*_*z*_ = 0.48) or touching (M ± SE = .58 ± 0.03, *t*(29) = 3.63, p < 0.001, *d*_*z*_ = 0.66). A main effect of posture was also present when there were two fingers in-between, F(2,58) = 7.16, p < 0.002, MSE = 0.013, η_p_^2^ = 0.20, caused by lower numerosity judgments when the fingers were touching (M ± SE = 1.29 ± 0.06) than when they were close (M ± SE = 1.38 ± 0.06, t(29) = 4.27, p < 0.0002, d_z_ = 0.78) or splayed (M ± SE = 1.39 ± 0.06, t(29) = 3.48, p < 0.002, d_z_ = 0.64).

As shown in Fig. 2D analysis of RTs revealed no main effect of posture, F(2,58) = 0.42, p > 0.66, MSE = 6893.290, η_p_^2^ = 0.01, nor interactions, F(4,116) = 0.78, p > 0.55, MSE = 4590.466, η_p_^2^ = 0.03, similarly to Experiment 1, supporting the evidence that the task is not easier when the fingers are splayed compared to when they are close or touching. Also for the localization task, there was a significant main effect of finger in-between, F(2,58) = 13.60, p < 0.0001, MSE = 17039.506, η_p_^2^ = 0.32, with RTs increasing with the number of fingers in-between. Moreover, response times were slower in Experiment 2 compared to Experiment 1, as shown by a between-experiments comparison, F(1,58) = 21.47, p < 0.0001, MSE = 803593, η_p_^2^ = 0.27. This effect may derive from several factors: first, in Experiment 2 participants have to mentally search the lexicon and organize an answer that will include two words; second, they have to decide which finger to report first, a completely arbitrary choice.

In contrast, in Experiment 1 they have to report only a number that represents the numerosity of the fingers in-between the two touched. The fact that in Experiment 2 participants used the fingers’ names to respond, could have required the use of an additional representation such as the so-called “body semantics”^6^, increasing the complexity of the mental processing. We acknowledge that this may be a possible limitation of the direct comparison between the two experiments. However, participants in Experiment 2 did not have difficulties in identifying the fingers as shown by the equal percentage of errors in the two experiments F(1,58) = 0.598, p = 0.44, MSE = 1506, η_p_^2^ = 0.01.

## Discussion

We examined in healthy humans whether structural body representations are modulated by changes in body posture using two classical tests of finger agnosia^3^. We hypothesized that if body structural representations are stable and do not vary as a function of posture, judged finger numerosity should be unaffected by changes in hand posture. In both tasks, however, the spatial distance between the fingers modulated perceived finger numerosity. Specifically, despite an overall underestimation of finger numerosity across conditions, judgements were higher when the fingers were splayed compared to when they were close or touching. However, this effect was present only when tactile stimuli were presented on non-adjacent fingers.

These results suggest that the body structural representations are not as fixed as commonly thought, but instead are updated as a function of the position of the fingers in external space, at least when non-adjacent fingers are stimulated. Therefore, body structural representations seem to be required only for judgments of body parts which are not directly adjacent. This dissociation can be attributed to the fact that normally, adjacent body parts such as the fingers do not change their relative position in space to each other, primarily due to the physical mechanical constraints. Therefore, sensory representations may be sufficient to track the relative position of the fingers that is “assumed” to be always the same (e.g., left middle finger stand on the left compared to the left index finger). In this respect, it has been shown that unusual postural configurations typically lead to conflict that, in turn, creates illusory percepts such as the Aristotle Illusion^20^, a circumstance which we will discuss below. We propose that a stable sensory representation based on anatomical coordinates is used when adjacent fingers are stimulated. Instead, differently from what was believed, our data show that less fixed body structural representations which take into account, at least to some extent, the external coordinates are used when non-adjacent fingers are stimulated. Furthermore, the fact that the localization task replicates exactly the results of the “in-between” test suggests that changes in finger posture alters the localisation of touch, rather than altering the representation of the fingers themselves.

Overall, our data show that body structural representations are not as fixed as previously thought and the way in which the fingers are represented varies as a function of the anatomical proximity between the stimulated fingers. In particular, identification of non-adjacent fingers occurs using at least in part an external reference frame, whereas identification of adjacent fingers occurs primarily using a reference frame based on anatomical coordinates.

### Tactile identification of adjacent fingers is not affected by changes in posture

Our results showed that tactile identification of adjacent fingers was not modulated by changes in finger posture. These results are compatible with the idea that identification of tactile stimuli on adjacent fingers relies on the use of anatomical coordinates in which the relative position between the fingers is not taken into account. In this respect, Schweizer and colleagues^21^,^22^ performed several studies investigating the pattern of tactile mislocalizations across fingers. They found that mislocalizations occur predominantly to fingers adjacent to the stimulated finger, reflecting the homuncular organization of the primary somatosensory cortex^21^. They suggested that the greater mislocalisation attributed to the neighbouring fingers compared to more distance fingers derives from the fact that adjacent digits have overlapping receptive fields^23^,^24^. Similarly, in a behavioural investigation using a double tactile simultaneous stimulation (DSS) paradigm, Tamè and colleagues^25^ asked participants to detect tactile stimuli at a pre-defined target finger that was stimulated alone or concurrently with another finger. They found interference effects when the distracting stimulation was on the non-homologous adjacent finger of the same hand, and when it was on the non-homologous finger of the opposite hand with respect to the target. Critically, when the spatial relationship between the hands in the external space was changed (i.e., one hand rotated upside down) the pattern of results at the within hand level (i.e., how much the target finger was masked by the stimulation of the adjacent finger) was not altered^25^.

Along the same lines, the so-called ‘Aristotle illusion’^20^ shows that when adjacent fingers are crossed the correct original localization of the fingers is maintained and not updated. A further study by Haggard and colleagues^26^, showed that changes in hand posture affect the identification of which hand was tactile stimulated, but not the simple detection of touches or identification of the stimulated fingers. The authors suggested that finger identification takes place at a low level of the tactile processing, namely based on skin coordinates of the somatotopic map, whereas identification of the hand occurrs at a later stage that takes into account postural information of the body. It is important to note that in all the studies we just described only adjacent fingers were stimulated. This is compatible with our results, and in particular, with the dissociation that we found in the identification process of adjacent (i.e., no postural modulation) vs. non-adjacent (postural modulation) fingers (see next section).

Overall, our data are in agreement with several lines of evidence in the literature showing that, under certain circumstances, representations of touch may not be susceptible to changes in body posture. This can be traced to the need of maintaining a stable representation of the structure of our body while we are moving in space or performing actions. Moreover, it can be due to the inaccurate assumption made by our brain that some spatial relationships between certain body parts (e.g., neighbouring fingers) can never be altered.

### Tactile identification of non-adjacent fingers is affected by changes in posture

Our results showed that tactile identification of the fingers, when stimuli were presented on non-adjacent fingers, was modulated by changes in finger posture. In particular, judgements of finger numerosity were increased when the fingers were splayed compared to when their fingers were touching or close. The tasks that we used are generally considered to be reliable measures of body structural representations^3^,^5^,^9^. Thus, this result suggests that BSRs are not as fixed as generally thought, but vary as a function of posture. This is in agreement with a substantial literature showing modulatory effects of posture in tactile localization on the fingers at different stages of the tactile representation processing. For instance, Overvliet and colleagues^27^, using von Frey hairs in a single finger stimulation task in which participants had to name the finger touched, have shown that the relative positions of the fingers influence tactile localization by reducing the number of mislocalizations when the fingers were splayed compared to when they were touching. This is compatible with our results when non-adjacent fingers were stimulated, though, in Overvliet and colleagues’ study, unlike the present work, a single tactile stimulus was always applied. Moreover, Tamè and colleagues^25^ found that when hand posture is altered, DSS interference remained unchanged within-hands, but became less consistent between hands. Therefore, this posture-dependent modulation indicates the adoption of spatial representations for touch, which take into account the overall structure of the body as well as its layout in space^25^.

In addition, Rusconi and colleagues^5^, investigated the effect of changes in hands’ posture using an inter-manual version of the classical “in-between” finger gnosis task. In their study participants decided whether the finger distance between two touches on one hand was the same as the finger distance between two touches on the other hand. Note that unlike our study, Rusconi and colleagues manipulated and stimulated the fingers of both hands. They used three types of conditions: “total homology” in which the same fingers were touched, “partial homology” in which one of the two fingers touched on the two hands was the same, and “no homology” in which both fingers touched on the two hands were different. They found that participants’ performance was affected by hands posture when homologous fingers of the two hands were stimulated, a condition that it is assume to adopt a sensory code based on anatomical coordinates. However, when partial or no homologous fingers were stimulated, the ones that are assumed to involve the BSRs, they did not find a modulation of the hands posture. The discrepancy between the results of Rusconi *et al*. and our work can be attributed by differences in the experimental design. In particular, we stimulated and varied the physical position of the fingers of the same hand, whereas Rusconi *et al*. stimulated the fingers and varied the position of the two hands. In this respect, it has been shown that representations and interactions of tactile stimuli on the fingers within and between the hands are coded using different types of processing^25^,^28^.

An example of how changes in fingers posture can alter body representations, compatible with our results, is provided by Longo^29^. In this study the author investigated how postural changes affect implicit body representations underlying position sense, which have been shown to be highly distorted^30^. Participants localised the knuckles and tips of each finger in external space in two postures, namely with the fingers touching or splayed. Spreading the fingers apart produced increases in the implicit representation of hand size, with no apparent effect on hand shape. Thus changes of internal hand posture produced rapid modulation of how the hand itself was represented^29^.

What drives the changes we observe in perceived finger numerosity when we stimulated non-adjacent fingers that are splayed? One possibility is that posture produces real-time modulation of somatotopic maps in somatosensory cortex. Previous studies have demonstrated rapid plasticity of primary^31^,^32^ and secondary^33^ somatosensory cortex. Hamada and Suzuki (2005), for example, found an increase in the distance between representations of the thumb and index finger in SII when the hand was open compared to when it was closed, suggesting that spreading the fingers made the representations of the digits more distinct. Such effects could potentially account for the present results if left posterior parietal representations of body structure interact dynamically with lower-level somatosensory representations. In this respect, a recent fMRI meta-analysis by Di Vita and colleagues^17^ found that non-action oriented body representations selectively activate the primary somatosensory cortex as well as the supramarginal gyrus, whereas action-oriented body representations such as the body schema showed selective activity for the primary motor cortex and the extrastriate body area (EBA)^17^.

Overall, our results demonstrate that structural body representations are not as fixed as commonly thought, but are modulated by the real-time posture of the body. This indicates that “online” and “offline” representations of the body^34^ are not fully distinct, but interact in intuitively surprising ways. Similar dynamic interactions are documented by a study of Craig^35^ in which he has shown how moving tactile stimuli on the fingers, though irrelevant to the task, determines the adoption of different reference frames. In his work the author used a temporal order judgement (TOJ) task with moving stimuli on the fingerpads. He presented a pattern of moving stimuli with different orientation and participants have to indicate which of the two tactile stimuli was presented first. In one experiment, he asked participants to perform the task with stimuli delivered on the same hand altering the position of the finger (i.e., index and middle fingers uncrossed vs. crossed), as in the classical Aristotle’s Illusion. Results showed that the TOJ bias, contrary to the Aristotle’s Illusion, did not vary as a function of the fingers posture. Instead, it remains stable suggesting that, in this particular circumstance, participants used an external reference frames to identify the stimuli on the fingers. Moreover, our results are compatible with a study of Brozzoli and colleagues^36^ showing that we can use both space- and body-based representations to represent numbers on our fingers. This is relevant for the present work considering that we asked participants to give numerical responses, at least in the first Experiment. Indeed, fingers are special body parts in terms of numbers as we learn to count on our fingers, and a digital representation of numbers is still present in adulthood^37^.

Finally, it is important to note that in the present study we investigated the body structural representations using tactile stimuli as inputs. Indeed, most previous studies have used visual input to investigate the properties of body structural representations^6^. Our approach may be of interest in helping to understand the properties of these representations using a different sensory system. Further, future studies may investigate whether using different type of inputs, such as visual or linguistic, can produce similar results we report for touch. This may be particularly useful in order to be able to develop specific rehabilitative strategies as a function of the type of body representations deficit.

## Conclusion

The results of the present work show that the representations used to identify tackily the fingers, can sometimes act as stable representations that code the relative position between the fingers regardless of postural changes. However, it can also dynamically adapt as a function of the postural changes occurring when fingers are moving in space. What it makes these representations stable rather than dynamic is the anatomical proximity of the fingers touched. Indeed, when adjacent fingers are stimulated the BSRs are not affected by changes in fingers posture, as it is instead, when stimuli occur on non-adjacent fingers. Most likely, the identification of tactile stimuli on adjacent fingers is occurring in a context in which anatomical coordinates dominate the spatial encoding, whereas when non-adjacent fingers are stimulated the reference frame that prevail is based on external reference frame coordinates. Research on body representations has historically focused on dissociating distinct classes of representations, such as between the body structural description, the body schema, and the body image. Our results underscore the importance of understanding not just how body representations differ, but also how they dynamically interact most likely to exert appropriate controlled actions.

## Methods

### Experiment 1: in-between test

#### Participants

Thirty people (mean ± SD = 30.6 ± 8.2 years; 16 females) participated. Participants reported normal or corrected to normal vision and normal touch. All participants were right-hand as assessed by the Edinburgh Handedness Inventory^38^; M = 90, range 50–100.

#### Apparatus and stimuli

Tactile stimuli were delivered on the fingers of the left hand using five solenoid tappers (8 mm in diameter; M&E Solve, UK) driven by a 9 V square wave. Our decision to stimulate the non-dominant hand was not especially motivated by strong theoretical considerations. However, we had a practical reason, stimulating the left hand was much easier with respect to our laboratory set-up and stimulators. The apparatus was controlled by means of a National Instrument I/O box (NI USB-6341) connected to a PC through a USB port. Tactile stimulation was delivered for 5 ms. All participants clearly perceived this stimulation when delivered in isolation to each finger before the experiment. To assure that when in operation the stimulators produced an equal force to the fingers a piezoelectric pressure sensor (MLT1010, AD Instruments, Dunedin, New Zealand) was used to measure the intensity of each tapper. Tactile stimulators were attached on the back of the fingers on the second phalanx centred with respect to the width and length of the finger using double-sided adhesive collars (ADD204 19 mm OD, 4 mm ID). The hands rested on the table aligned with the participant’s body midline in a comfortable position (Fig. 1A). In this way, the stimulators exerted a similar pressure on all body parts.

Vision of the hand was prevented throughout by means of a sheet of black cardboard, placed horizontally on a structure fixed to the table, on top of the hands. Participants responded vocally by speaking into a microphone positioned in front of their mouth. Their vocal response was saved to a.wav file for offline coding. The start of the audio recording was time-locked to the tactile stimulation to allow computation of vocal reaction time. To facilitate coding, the experimenter entered the participant’s response into the computer. Stimulus presentation and response collection were controlled by a custom program written using MATLAB R2013b (Mathworks, Natick, MA) and the Psychtoolbox libraries^39^. Throughout the experiment, white noise was presented over closed-ear headphones (Sennheiser HD 439 Audio Headphones) to mask any sounds made by the tactile stimulators.

#### Procedure

Participants were instructed to keep their gaze directed towards a black sticker on the wall in front of them aligned with their body midline to keep head^40^ and gaze position^41^,^42^ constant. At the beginning of each trial a pair of tactile stimuli were presented simultaneously after a variable interval, ranging from 0 to 1000 ms. By stimulating different pairs of fingers, we created situations in which there were zero (i.e., thumb-index, index-middle, middle-ring, ring-little), one (i.e., thumb-middle, index-ring, ring-little), or two (i.e., thumb-ring, index-little) fingers in-between the stimulated fingers. Participants responded verbally, as quickly and accurately as possible judging how many unstimulated fingers there were in-between the two touched fingers. If the participant did not respond after 3000 ms, a new trial began. The experimenter remained in the room throughout the session to ensure that participants complied with the instructions and to record the responses. No feedback about performance was provided. There were six blocks, two of each posture, presented counterbalanced in ABCCBA sequence, with the first three conditions counterbalanced across participants. The number of trials was balanced between the different fingers paired stimulated (i.e., “Zero”, “One” or “Two” fingers) with 24 trials each, resulting in 72 trials per block and a total of 432 trials. Given the purpose of the study, we decided to have the same number of trials for each fingers pairs stimulated (i.e., “Zero”, “One” or “Two” fingers in-between), which results in a greater number of fingers combinations for the conditions with no or fewer fingers in-between. They were allowed short breaks between blocks.

#### Data Analysis

To obtain an index of under- and over-estimation of the in-between fingers touched, we averaged responses based on the actual number of fingers in-between (i.e., Zero, One, Two) and the spatial arrangement of the fingers (Touching, Close, Splayed). The average response numerosities and reaction times (RTs) were entered into separate two-way Analyses of Variance (ANOVAs) with posture (Touching, Close, Splayed) and fingers in-between (Zero, One, Two) as within-participant factors. RTs were extracted from the vocal responses by using a custom Matlab script. The Matlab function used to record the vocal responses produced a temporal delay which we measured in a separate session to have a mean of 404 ms and a SD of 15 ms. As this fluctuation was randomly distributed across trials and conditions, we have added 404 ms to the mean obtained in each condition. Moreover, the data for each trial were visual inspected in order to exclude trials in which the response fell outside the temporal interval (i.e., greater than 3 seconds) or in which no response was made. RTs were computed on all participants’ responses, as it was for the number of judged fingers in-between.

### Experiment 2: localization task

#### Participants

Thirty people (mean ± SD = 33.4 ± 11.9 years; 18 females) participated. Participants reported normal or corrected to normal vision and normal touch. All participants were right-hand as assessed by the Edinburgh Handedness Inventory^38^; M = 90, range 26–100.

#### Procedure

Procedures were identical to Experiment 1, with the following exception. Participants were asked to judge which fingers were actually touched (Fig. 2A), differently from Experiment 1 in which they have to report the number of unstimulated fingers in-between the two touched fingers.

#### Data Analysis

In Experiment 1 the judged number of fingers in-between was obtained directly from the participant’s response. In this experiment a similar measure was obtained indirectly by calculating the number of fingers in between the two fingers that the participant judged to have been stimulated. For example, if the participant reported that the index and little fingers were touched, we treated this as a judgment of two fingers in-between (i.e., the middle and ring fingers). RTs were calculated taking into account the time of the response from the first finger named of the two stimulated fingers.

#### Ethics

Participants gave their informed consent prior to participation. The study was approved by the local ethical review committee at Department of Psychological Sciences, Birkbeck College, University of London and was carried out according to the principles of the 1964 Declaration of Helsinki.

## Additional Information

**How to cite this article**: Tamè, L. *et al*. Finger posture modulates structural body representations. *Sci. Rep.* **7**, 43019; doi: 10.1038/srep43019 (2017).

**Publisher's note:** Springer Nature remains neutral with regard to jurisdictional claims in published maps and institutional affiliations.
